# Impact of early Kasai portoenterostomy on short-term outcomes of biliary atresia: A systematic review and meta-analysis

**DOI:** 10.3389/fsurg.2022.924506

**Published:** 2022-09-01

**Authors:** Changzhen Yang, Meng Ke, Yan Zhou, Hang Xu, Mei Diao, Long Li

**Affiliations:** ^1^ Department of Pediatric Surgery, Capital Institute of Pediatrics, Beijing, China; ^2^ Graduate School of Peking Union Medical College, Chinese Academy of Medical Sciences, Beijing, China; ^3^ Research Unit of Minimally Invasive Pediatric Surgery on Diagnosis and Treatment (2021RU015), Chinese Academy of Medical Sciences, Beijing, China; ^4^ State Key Laboratory of Cardiovascular Disease, Fuwai Hospital, National Center for Cardiovascular Diseases, Chinese Academy of Medical Sciences and Peking Union Medical College, Beijing, China

**Keywords:** Kasai portoenterostomy, biliary atresia, age at operation, short-term outcomes, meta-analysis

## Abstract

**Background:**

Good outcomes of biliary atresia (BA) are conventionally achieved after early Kasai portoenterostomy (KP). However, in some recent pieces of literature, there are discrepancies in the influence of age in Kasai procedure on postoperative short-term prognosis. This meta-analysis aims to evaluate the effects of earlier KP on short-term surgical prognosis of BA and clarify these discrepancies in recent studies.

**Methods:**

To identify related studies, PubMed, Embase, Web of Science, Cochrane, and the Chinese National Knowledge Infrastructure database were searched up to March 2022. Data for the impact of age at KP on clinical prognosis were extracted, including jaundice clearance rate (JCR) and native liver survival rate (NLSR).

**Results:**

A total of 14 articles were included in the present study, which involve a total of 3,276 patients with BA who underwent Kasai procedure. Compared with patients older than 91 days of age, patients 90 days of age or younger exhibited significantly better JCR [odds ratio (OR), 3.05; 95% confidence interval (CI), 2.23–4.17; *P* < .001] and a more favorable NLSR (OR, 1.72; 95% CI, 1.37–2.15; *P* < .001). The NLSR of patients younger than 60 days of age was significantly higher than those of patients from 61 to 90 days of age (OR, 1.41; 95% CI, 1.18–1.68; *P* < .001). There was no significant difference in JCRs between patients aged 60 days of age or younger and those aged 61–90 days of age (OR, 1.31; 95% CI, 0.95–1.81; *P* = 0.10). Among patients 30 days of age or younger, 31–45 days of age, and 46–60 days of age, there were also no significant differences in JCR.

**Conclusion:**

A significantly better short-term JCR and NLSRs were achieved among patients with BA treated using a KP procedure at ≤90 days of age compared with those treated at >90 days of age. There was no further improvement in the short-term JCR when the procedure was performed at ≤60 days compared with those treated at 61–90 days of age. However, treatment at ≤60 days of age was associated with a significant improvement in NLSR. Therefore, the timing of KP does exert an important effect on short-term clinical outcomes of patients with BA.

## Introduction

Biliary atresia (BA) is a serious neonatal disease caused by intrahepatic or extrahepatic bile duct occlusion. Its etiology and pathogenesis are controversial (1). The incidence of BA ranges from 1 in 5,000 to 1 in 19,000 live births (2, 3). BA patients can only survive with a successful surgical operation. Kasai portoenterostomy (KP) is currently recognized as the primary procedure for restoring bile flow and preserving the native liver of BA patients (4–7). There are several indicators that may influence the outcomes of BA patients after KP, including various etiologies, association anomalies, degrees of native liver damage, type of obstruction, steroid regimen, and age at the KP (8). Among these, age at the KP has gained the attention of many studies that have indicated that earlier KP is associated with more favorable outcomes (9–11). These favorable outcomes are usually associated with efficient bile drainage and mild liver pathological changes (11). However, some recent studies have indicated that early KP is not associated with good BA outcomes (12, 13). Others considered that KP is associated with worse outcomes for younger BA patients (14, 15). However, these discrepancies might attribute to the small sample sizes involved in those studies. Therefore, it is necessary to perform meta-analyses to reassess the effect of early KP on jaundice clearance rate (JCR) and native liver survival rate (NLSR). It is also necessary to perform subgroup analyses based on the BA type and indicators of the final diagnosis. The present meta-analysis extracted data from published studies to evaluate the impact of age at the time of KP on the outcomes and to clarify the existing discrepancies reported by recent studies.

## Methods

### Search strategy and study identification

Our systematic review and meta-analysis were conducted according to the Preferred Instrument for Systematic Reviews and Meta-Analysis (PRISMA) guidelines (16). The protocol was defined and followed. The protocol was not registered, but without a similar registered protocol on online registry sites. The PICO (Participants, Intervention, Comparison, and Outcome) framework was used as follows: participants, patients with BA treated with KP; intervention, younger age at the time of KP compared to older age; and outcomes, JCR and NLSR. A systematic search of PubMed, Embase, Web of Science, Cochrane, and Chinese National Knowledge Infrastructure (CNKI) databases was performed up to March 2022. The medical subject terms and keywords were as follows: “Portoenterostomy, Hepatic”, “Biliary Atresia”, and “Age/Day/Days”. The detailed search strategy can be found in Supplementary Material 1.

### Eligibility criteria and data extraction

The inclusion criteria were as follows: population comprising patients younger than 18 years with BA; intervention for and comparison of patients who underwent KP at different ages; the study reported the NLSR at 2 years and/or the JCR at 3 or 6 months; and Newcastle–Ottawa Scale (NOS) score ≥6. Meeting abstracts, case reports, reviews, meta-analyses, animal studies, and duplicate publications were excluded. The following information was independently extracted by two researchers: first author; publication year; study design type; sample size; age at surgery; NLSR; JCR; BA type; and steroid regimen. Any disagreements regarding the qualifications and data collection were resolved through discussions with the corresponding authors and other authors.

### Statistical methods and exploration of heterogeneity

Statistical analyses were conducted by Stata/SE 12.0. Odds ratio (OR) and 95% confidence interval (CI) were calculated. Statistical significance was judged by *P* < .05. Statistical heterogeneity was evaluated using the Cochrane *Q* test and *I*^2^ values; *P* > 0.1 and *I*^2^ < 50% were considered low heterogeneity, indicating that a fixed effect model could be used. Otherwise, a random effect model was used when the high heterogeneity was considered (*P* < 0.1 and *I*^2^ > 50%) (17). Subgroup analyses were conducted based on BA type (multiple types vs. single-type), final prognostic indicators (JCR at 3 vs. 6 months), and region of the study (China vs. non-China). Subgroup analyses were used to identify potential sources of heterogeneity and to explore the potential influence of known factors on outcomes. Additionally, a sensitivity analysis was conducted to identify the reliability and validity of this study.

### Quality assessment and publication bias

Two independent reviewers rated the quality of the included studies using the NOS for cohort studies. Studies with NOS scores ≥6 could be included in the meta-analysis. The NOS checklist is listed in Supplementary Material 6. Because of the number of articles included in this study, a publication bias analysis was not necessary.

## Results

### Search results and study characteristics

**Figure 1:**
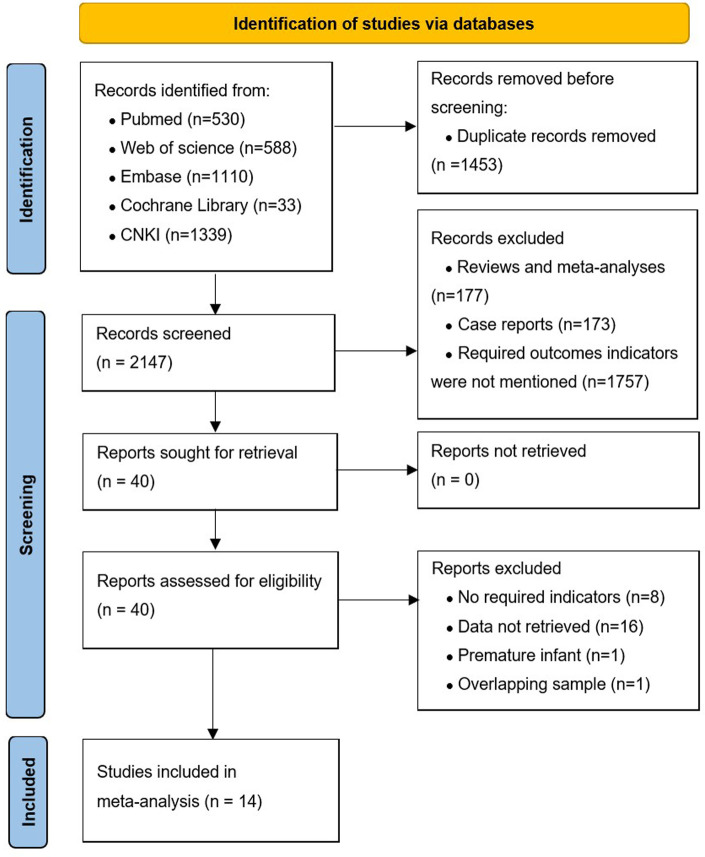
Flowchart of literature search and study selection.

Figure 1 describes the literature search and screening process. Our search identified a total of 3,600 records, as follows: PubMed (*n* = 530); Web of Science (*n* = 588); Embase (*n* = 1110); Cochrane Library (*n* = 33); and CNKI (*n* = 1,339). There were 2,147 articles retained after Endnote and manual checking were performed to identify duplicates. Then, these 2,147 articles were further screened according to the titles and abstracts. There were 1,757 articles without relevant outcome indicators, 177 reviews and meta-analyses, and 173 case analyses. Therefore, the remaining 14 articles (12, 13, 18–29) (3,276 patients with BA) were included in the meta-analysis between 2009 and 2021. All included studies were cohort studies. Four studies included patients with single-type (type III) BA. Ten studies included patients with multiple BA types, including type I, II, and III BA (30). A total of nine studies reported that a steroid regimen was used for all included patients. Five studies reported that a steroid regimen was used for only some patients or a steroid regimen was not mentioned. The characteristics of the studies are shown in Table 1.

**Table 1:** Characteristics of the reports included in the meta-analysis.

Author	Year	Study type	Sample size	Age at KP	Native liver survival rate	Jaundice clearance rate	Type of BA	Steroid regimen	NOS score
Serinet et al. (18)	2009	Cohort	352	≤30 ds, 31–45 ds, 46–60 ds	35/59, 79/131, 89/162	NA	Multi-types	NA	8
				61–75 ds, 76–90 ds, ≥90 ds	82/159, 43/90, 31/83				
Nio et al. (20)	2010	Cohort	242	<60 ds, 61–90 ds, 91–120 ds	NA	49/67, 65/114, 15/38	III-type	NA	7
				<121 ds		3/23			
Ferreira et al. (19)	2010	Cohort	117	<60 ds, 60–90 ds, >90 ds	NA	9/20, 20/51, 10/46	Multi-types	Not all	6
Gong et al. (12)	2012	Cohort	452	<60 ds, 60–90 ds, >90 ds	58/146, 93/222, 30/84	NA	III-type	All	6
Lin et al. (21)	2012	Cohort	151	≤60 ds, 61–90 ds, >90 ds	NA	15/28, 43/83, 16/40	Multi-types	All	6
Li et al. (22)	2015	Cohort	99	<60 ds, 60–90 ds, >90 ds	NA	11/18, 22/40, 11/41	Multi-types	All	6
Xu et al. (23)	2016	Cohort	84	≤60 ds, 61–90 ds, >90 ds	19/27, 14/32, 10/25	20/27, 17/32, 12/25	Multi-types	All	6
Song et al. (24)	2017	Cohort	476	≤60 ds, 61–90 ds, >90 ds	89/158, 117/224, 39/94	NA	Multi-types	NA	6
Sun et al. (25)	2017	Cohort	91	<60 ds, 60–89 ds, ≥90 ds	19/34, 21/47, 1/10	NA	Multi-types	All	6
Yang et al. (26)	2018	Cohort	75	≤60 ds, 61–90 ds, >90 ds	21/28, 23/39, 2/8	NA	Multi-types	All	6
Zhou et al. (13)	2019	Cohort	220	≤30 ds, 31–45 ds, 46–60 ds	NA	6/10, 24/49, 31/65	III-type	All	7
				61–90 ds, ≥91 ds		40/76, 10/20			
Ryuji et al. (27)	2020	Cohort	224	≤30 ds, 31–45 ds, 46–60 ds	NA	10/10, 11/17, 49/59	III-type	NA	8
				<60 ds		90/138			
Wu et al. (28)	2021	Cohort	124	<90 ds, ≥90 ds	42/109, 4/15	NA	Multi-types	All	6
Zhao et al. (29)	2021	Cohort	569	≤45 ds, 46–60 ds, 61–75 ds	18/44, 74/165, 71/184	NA	Multi-types	All	7
				76–90 ds, <90 ds	31/92, 25/84				

NA, not available; ds, days; NOS, Newcastle–Ottawa Scale; KP, Kasai portoenterostomy; BA, biliary atresia; Cohort, Cohort study; Multi-types: multiple types patients (I, II, and III-types), All: all the patients received steroid regimen, Not all: not all the patients received steroid regimen.

### Jaundice clearance rate

#### Age 90 days or younger compared to age older than 90 days

**Figure 2:**
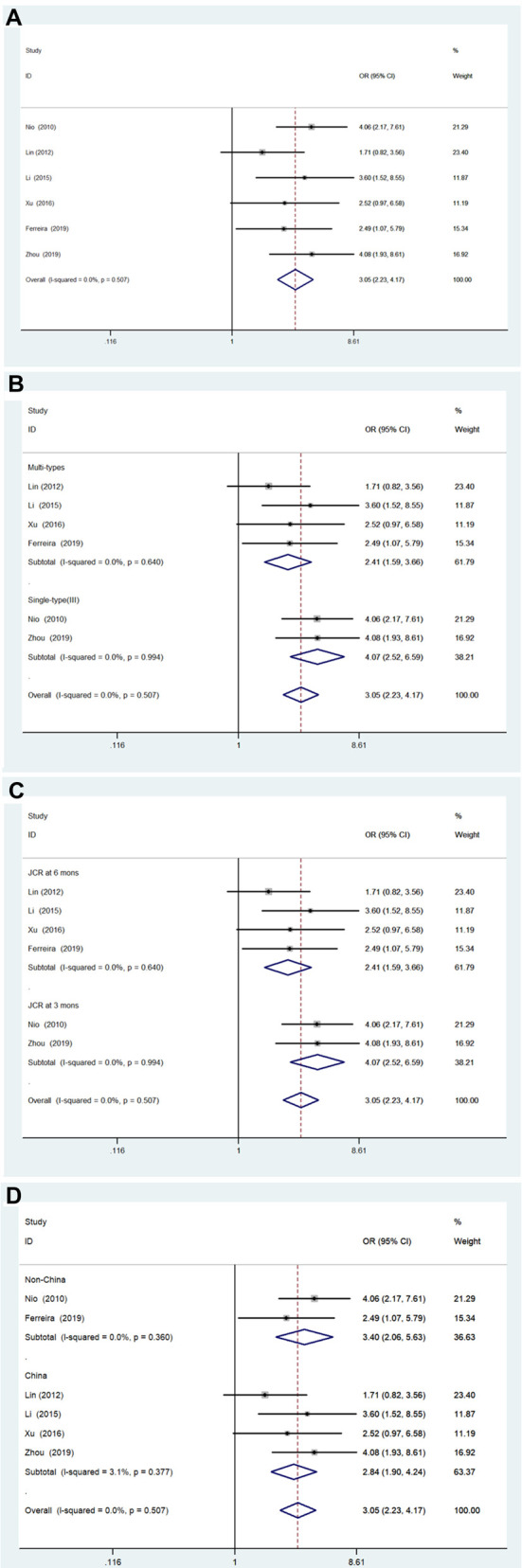
Forest plot for JCR of ≤90 days vs. >90 days group and results of subgroup analyses. (**A**) Forest plot for JCR. (**B**) Subgroup analysis based on biliary atresia type. (**C**) Subgroup analysis based on JCR at 3 vs. 6 months. (**D**) Subgroup analysis based on region. JCR, jaundice clearance rate.

Figure 2A shows the results of the pooled associations between the two groups. There were six studies (941 patients) (13, 19–23) included; there was no significant heterogeneity (*I*^2 ^= 0.0%; *P* = 0.507). The pooled OR was 3.05 (95% CI, 2.23–4.17; *P* < .001) using a fixed effects model analysis, suggesting that patients 90 days of age or younger could achieve a higher JCR. Furthermore, the results of the subgroup analyses are shown in Figures 2B–D, respectively. In the subgroup analysis stratified by BA type, the pooled OR of Multiple types was 2.41 (95% CI, 1.59–3.66; *P* < .001), and the pooled OR of single-type (type III) was 4.07 (95% CI, 2.52–6.59; *P* < .001). In the subgroup analysis based on final diagnosis indicators, the pooled OR of JCRs in 6 months group was 2.41 (95% CI, 1.59–3.66; *P* < .001), and the pooled OR of JCRs in 6 months group was 4.07 (95% CI, 2.52–6.59; *P* < .001). Furthermore, in the subgroup analysis based on region, the pooled OR of the China group was 2.84 (95% CI, 1.90–4.24; *P* < .001), and the pooled OR of the Non-China group was 3.40 (95% CI, 2.06–5.63; *P* < .001). The results of the subgroup analyses indicated that the three variables did not significantly influence the overall results of the studies.

#### Age 60 days or younger compared to age 61–90 days

**Figure 3:**
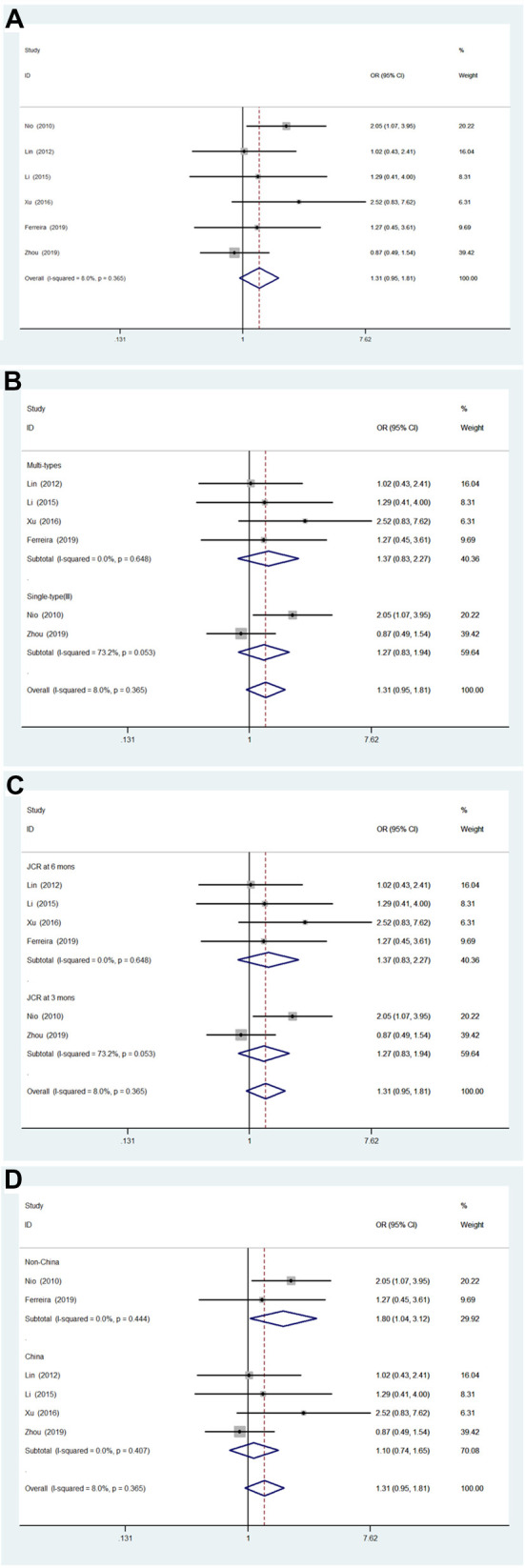
Forest plot for JCR of ≤60 days vs. 61–90 days group and results of subgroup analyses. (**A**) Forest plot for JCR. (**B**) Subgroup analysis based on biliary atresia type. (**C**) Subgroup analysis based on JCR at 3 vs. 6 months. (**D**) Subgroup analysis based on region. JCR, jaundice clearance rate.

A total of six studies (678 patients) (13, 19–23) were included (Figure 3). The BA type and JCRs at 3 or 6 months were also considered covariates of the subgroup. Among the six studies, heterogeneity was not significant (*I*^2 ^= 8.0%; *P* = 0.365), and significant heterogeneity was not found in the subgroup analysis (Figure 3A). The pooled OR of the six articles was 1.31 (95% CI, 0.95–1.81; *P* = 0.10) using a fixed effects model analysis, indicating that there was no significant difference in JCRs between groups. Furthermore, the pooled OR of the multiple types group was 1.37 (95% CI, 0.83–2.27; *P* = 0.223), and the pooled OR of the single-type (type III) group was 1.27 (95% CI, 0.83–1.94; *P* = 0.266). And the pooled OR of the JCR in 6 months group was 1.37 (95% CI, 0.83–2.27; *P* = 0.223), and the pooled OR of the JCR in 3 months group was 1.27 (95% CI, 0.83–1.94; *P* = 0.266). In subgroup analysis based on region, the pooled OR of the China group was 1.10 (95% CI, 0.74–1.65; *P* = 0.635), and the pooled OR of the Non-China group was 1.80 (95% CI, 1.04–3.12; *P* = .037), indicating that the statistically significant associations were found in only the non-China group.

#### Age 30 days or younger compared to age 31–45 days compared to age 46–60 days

**Figure 4:**
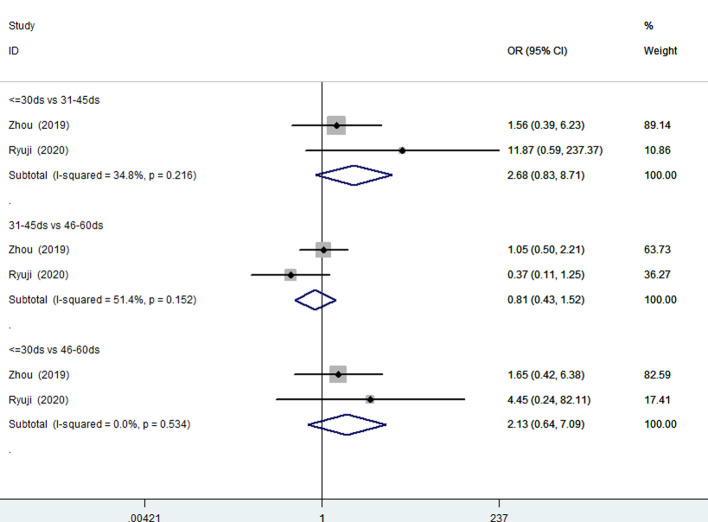
Forest plots for JCR of ≤30 days vs. 31–45 days, 31–45 days vs. 46–60 days, ≤30 days vs. 46–60 days. JCR, jaundice clearance rate.

Two studies (210 patients) (13, 27) involved patients 30 days of age or younger compared with patients 31–45 days of age, patients 31–45 days of age compared with patients 46–60 days of age, and patients 30 days of age or younger compared with patients 46–60 days of age were included. There was no significant heterogeneity (Figure 4). A fixed effects model analysis yielded pooled ORs of 2.68 (95% CI, 0.83–1.90; *P* = 0.10), 0.81 (95% CI, 0.43–1.50; *P* = 0.505), and 2.13 (95% CI, 0.64–7.09; *P* = 0.216), suggesting that younger age at the time of KP did not improve the JCRs of patients 60 days of age or younger.

### Native liver survival rate

#### Age 90 days or younger compared to age older than 90 days

**Figure 5:**
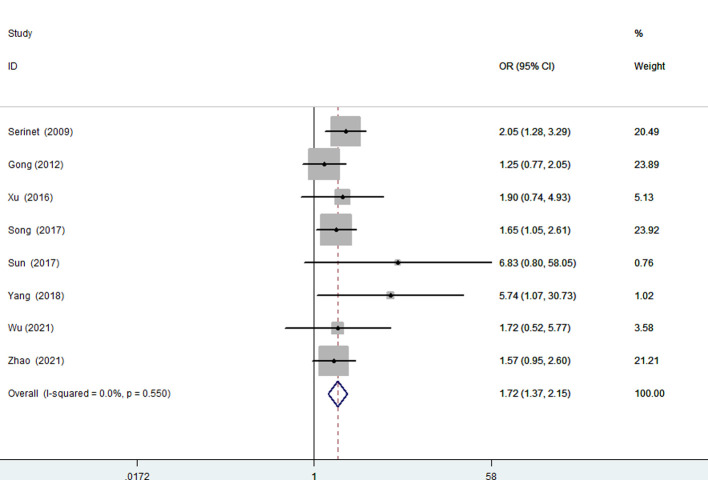
Forest plot for NLSR of ≤90 days vs. >90 days group. NLSR, native liver survival rate.

Eight articles were included (2,554 patients) (12, 18, 23–26, 28, 29), and there was no significant evidence of heterogeneity among them (*I*^2 ^= 0.0%; *P* = 0.550). The results of the fixed effects model showed that patients 90 days of age or younger can achieve a higher NLSR (OR, 1.72; 95% CI, 1.37–2.15; *P* < .001) (Figure 5).

#### Age 60 days or younger compared to age 61–90 days

**Figure 6:**
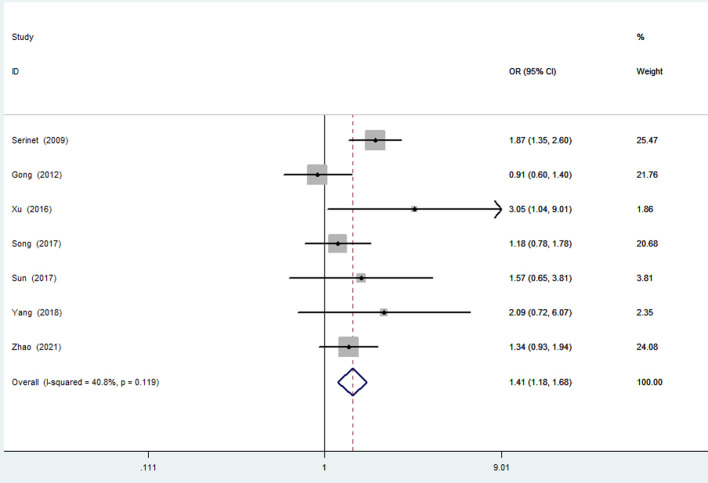
Forest plot for NLSR of ≤60 days vs. 61–90 days group. NLSR, native liver survival rate.

Seven studies (2,043 patients) (12, 18, 23–26, 29) reported the NLSRs of patients 60 days of age or younger and those of patients 61–90 days of age. Statistical heterogeneity was not found in the studies (*I*^2 ^= 40.8%; *P* = 0.119). A fixed effects model analysis was conducted to yield a pooled OR of 1.41 (95% CI, 1.18–1.68), and the difference was statistically significant (*P* < .001), suggesting that patients 60 days of age or younger can achieve a higher NLSR than patients 61–90 days of age (Figure 6).

### Sensitivity analysis

**Figure 7:**
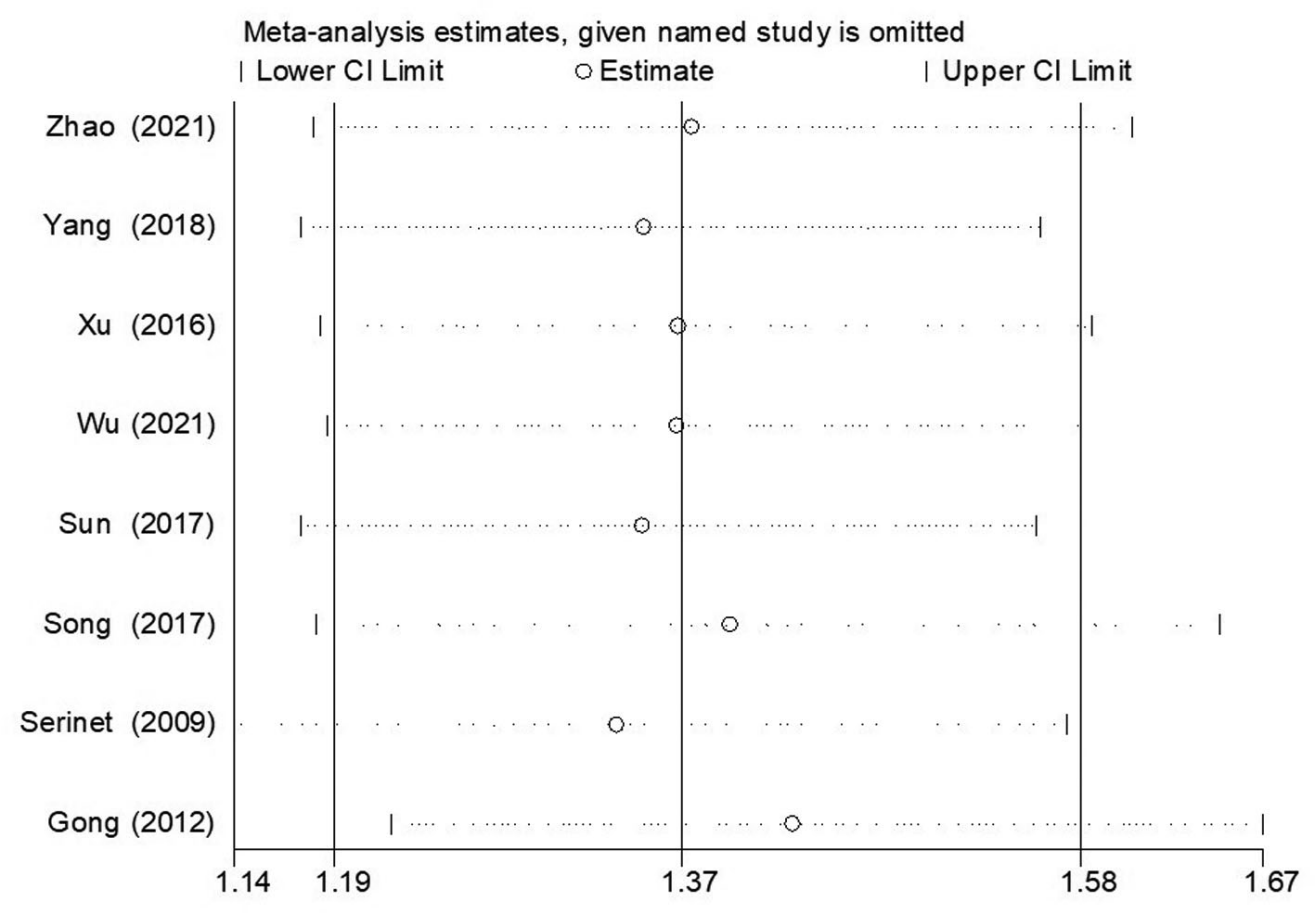
The result of sensitivity analysis regarding the assessment of NLSR of ≤90 days vs. >90 days group. NLSR, native liver survival rate.

The sensitivity analysis of NLSR and JCR was performed using Stata/SE 12.0, and the results indicated that our results were credible and stable. As shown in Figure 7, the sensitivity analysis for the NLSR of patients younger than 90 days and patients older than 91 days indicated that the result cannot be substantially changed by any one study, consistent with other groups (Supplementary Material 5).

## Discussion

With the development of diagnostic approaches, more and more BA patients have been confirmed and treated with early KP. Some researchers have reported that better JCR and NLSR are achieved after early KP (9–11) because of the better bile drainage, milder liver damage, and less cholestasis after early KP. Some studies have reported conflicting results (12–15); however, the exact cause of these discrepancies is still unclear. Zhou et al. (13) investigated the outcomes of 220 type III BA patients and found that age at the time of KP was not associated with JCR and NLSR, consistent with the studies of Gong et al. (12) and Riccardo et al. (31). Gong et al. (12) considered that patients with ductal plate malformation and other associated anomalies tended to have an early-onset BA and early KP. Older patients who underwent KP were more likely to have BA caused by perinatal infections (such as cytomegalovirus IgM BA), milder bile duct malformation, and more severe cholestasis in the upper bile ducts. Therefore, the large pressure gap induces quicker bilirubin excretion and comparable or even more prominent jaundice clearance after KP compared to those of patients who underwent early KP (12, 14). This speculation might be a probable explanation for our results that indicated no statistical differences in JCRs of patients younger than 60 days of age and patients 61–90 days of age. Furthermore, the study by Riccardo et al. (31) indicated that biliary atresia splenic malformation (BASM) was associated with worse NLSRs, which might occur regardless of the impact of age at KP. However, the study by Wong et al. (15) excluded patients with ductal plate malformation and other associated anomalies and concluded that the lowest postoperative bilirubin levels occurred in patients 61–80 days of age, attributing to better anastomosis of the portoenterostomy in BA patients 61–80 days of age. Additionally, Kyong et al. (32) reported that the impact of age at KP on postoperative outcomes became less significant with proficiency in surgery, increased center experience, and improved care for chronic liver disease.

In the present study, our results indicated that early KP may improve JCR at 3 or 6 months and NLSR at 2 years, consistent with the results of previous studies (9–11). But there was no significant difference was found in the JCRs of patients 90 days of age or younger, especially among patients younger than 30 days of age, patients 31–45 days of age, and patients 45–60 days of age, and the results were inconsistent with the results of Ryuji et al. (27). Furthermore, subgroup analyses for potential factors that might impact the outcomes of BA patients was conducted. Conventionally, the outcomes of type III BA were considered inferior to the outcomes of type I and type II BA (26); therefore, the included studies were divided into multiple types BA group and single-type BA group (type III). The results indicated that these subgroups had no significant effect on the overall outcomes, which might be attributed to the fact that the proportion of type III BA cases was largest (82%–90%) (19, 21–23) in the multiple types group. We also found differences in the definition of jaundice clearance. The subgroup analysis of JCRs at 3 months and JCRs at 6 months indicated that the differences in final outcome indicators did not affect the overall results. With regards to region, our subgroup analysis identified a better effect of non-China studies in improving the JCR for the ≤60 days compared to the 61–90 days age groups. A previous study in New Zealand (33) indicated that ethnicity may affect clinical outcomes, with a better NLSR reported for patients of Māori than European ethnicity. Hence, we conjecture that our finding of a benefit of non-China studies might reflect a prevalence of ductal plate malformation and other associated anomalies in the non-Chinese ethnicity, for whom earlier KP would be of benefit. By comparison, BA among patients of China was more likely to be caused by perinatal infections, for whom JCR can be improved with later KP, as previously described. Additionally, the results also could be due to the fact that postoperative adjuvant treatment protocols and economic circumstances varied greatly by country.

Furthermore, the various etiologies, association anomalies, postoperative steroid treatment, and operative approach (8), which might be associated with the outcomes should be considered to determine the credibility of the results of the present meta-analysis. Although some studies (34, 35) considered that postoperative steroid treatment might improve the bile drainage and native liver survival of BA patients, no sufficient evidence exists. In the present article, most of the included studies mentioned that all people had received postoperative steroid treatment, except the study by Ferreira et al. (19). Additionally, the effects of the operative approach (laparoscopic portoenterostomy vs. open portoenterostomy) (36) on the BA outcomes are also controversial. Therefore, subgroup analyses of adjuvant steroid treatment and operative approaches were not conducted. Furthermore, syndromic BA and associated extrahepatic malformations were often regarded as a certain factor associated with a poor prognosis, particularly the subset with BASM according to the Davenport BA classification (4). According to the study by Ryuji (27), the impact of age at KP might be less significant for certain subsets with associated anomalies. However, the accurate data regarding associated anomalies were rarely mentioned and insufficient for subgroup analysis of our included studies during this meta-analysis. In addition, BASM has a much lower incidence in Asian countries (37, 38), which might not be an independent risk factor for short-term BA outcomes (27). During the present meta-analysis, the majority (79%) of the included studies reported populations of Asian countries, and there was no statistical heterogeneity among the included studies, suggesting that our results were credible. In addition, a sensitivity analysis also showed the stability and reliability of the results of the meta-analysis, but further studies regarding the impact of various etiologies and association anomalies on the disease process and outcomes of BA are warranted.

Our meta-analysis had some limitations. The absence of randomized controlled trials was one of these limitations. Another limitation was that some studies were not included because of the differences in the age ranges of the patient groups, particularly patients younger than 60 days of age. More accurate groupings of patients, such as patients younger than 30 days of age, patients from 31 to 45 days of age, and patients from 46 to 60 days of age, should be studied. In addition, factors other than age need to be studied as they likely will have a prognostic impact, including postoperative adjuvant steroid regimens, including the initial dosage, administration methods, steroid type, and duration (34, 35); use of other adjuvant therapies, such as ursodeoxycholic acid, antibiotics, and fat-soluble vitamins (39–41); open vs. laparoscopy surgery (36); associated comorbidities or complications, such as thrombocytopenia and consumptive coagulopathy (42, 43); and various duration of follow-up. Data from the studies included were insufficient to conduct subgroup analyses in our meta-analysis. Thus, further studies are warranted.

## Conclusion

Our results indicate that KP performed earlier, at ≤90 days of age, can achieve more favorable short-term outcomes in terms of NLSR and JCR. Moreover, while KP performed at ≤60 days of age does not significantly improve JCR, there was a significant improvement in short-term NLSR. We note that although age at the time of KP is a significant prognostic factor, other factors can influence the outcomes of BA surgery. Further and long-term follow-up studies for outcomes of BA patients who underwent KP at 60 days of age or younger are warranted.

## Data Availability

The original contributions presented in the study are included in the article/**Supplementary Material**, further inquiries can be directed to the corresponding author/s.
