# Supplementary material for: Impact of early Kasai portoenterostomy on short-term outcomes of biliary atresia: A systematic review and meta-analysis
Source: Front Surg. 2022 Sep 1;9:924506. doi: 10.3389/fsurg.2022.924506 (PMC9475174; doi:10.3389/fsurg.2022.924506)
Supplement: Supplementary file 1 [file Data_Sheet_1_v1.pdf]

Search Strategy:

Pubmed: a total of 530 articles

(((age[Title/Abstract]) OR (day[Title/Abstract])) OR (surgery age[Title/Abstract]))) AND ((((((((("Portoenterostomy, Hepatic"[Mesh]) OR (Portoenterostomy, Hepatic[Title/Abstract])) OR (Hepatic Portoenterostomy[Title/Abstract])) OR (Hepatic Portoenterostomies[Title/Abstract])) OR (Portoenterostomies, Hepatic[Title/Abstract])) OR (Hepatoportoenterostomy[Title/Abstract])) OR (Hepatoportoenterostomies[Title/Abstract])) OR (Kasai Procedure[Title/Abstract]))) AND (((((((((("Biliary Atresia"[Mesh]) OR (biliary atresia[Title/Abstract])) OR (Atresia, Biliary[Title/Abstract])) OR (Intrahepatic Biliary Atresia[Title/Abstract])) OR (Atresia, Intrahepatic Biliary[Title/Abstract])) OR (Biliary Atresia, Intrahepatic[Title/Abstract])) OR (Biliary Atresia, Extrahepatic[Title/Abstract])) OR (Atresia, Extrahepatic Biliary[Title/Abstract])) OR (Extrahepatic Biliary Atresia[Title/Abstract])) OR (Idiopathic Extrahepatic Biliary Atresia[Title/Abstract])) OR (Familial Extrahepatic Biliary Atresia[Title/Abstract])))

Web of science: a total of 588 articles

((TS=(Portoenterostomy, Hepatic) OR AB=(Portoenterostomy, Hepatic OR Hepatic Portoenterostomy OR Hepatic Portoenterostomies OR Portoenterostomies, Hepatic OR Hepatoportoenterostomy OR Hepatoportoenterostomies OR Kasai Procedure)) AND (TS=(Biliary Atresia) OR AB=(biliary atresia OR Atresia, Biliary OR Intrahepatic Biliary Atresia OR Atresia, Intrahepatic Biliary OR Biliary Atresia, Intrahepatic OR Biliary Atresia, Extrahepatic OR Atresia, Extrahepatic Biliary OR Extrahepatic Biliary Atresia OR Idiopathic Extrahepatic Biliary Atresia OR Familial Extrahepatic Biliary Atresia))) AND (((AB=(age)) OR AB=(surgery age)) OR AB=(day))

Embase: a total of 1110 articles

No.	Query Results	Results
#8.	#6 AND #7	1,110
#7.	#4 AND #5	2,183
#6.	#1 OR #2 OR #3	5,533,057
#5.	'portoenterostomy, hepatic'/exp OR 'portoenterostomy, hepatic' OR (('portoenterostomy,'/exp OR portoenterostomy,) AND hepatic) OR 'hepatic portoenterostomy'/exp OR 'hepatic portoenterostomy' OR (hepatic AND ('portoenterostomy'/exp OR portoenterostomy)) OR 'hepatic portoenterostomies' OR (hepatic AND portoenterostomies) OR 'portoenterostomies, hepatic' OR (portoenterostomies, AND hepatic) OR 'hepatoportoenterostomy'/exp OR hepatoportoenterostomy OR hepatoportoenterostomies OR 'kasai procedure'/exp OR 'kasai procedure' OR (kasai AND ('procedure'/exp OR procedure))	11,580
#4.	'biliary atresia':ab,ti OR 'atresia, biliary':ab,ti OR 'intrahepatic biliary atresia':ab,ti OR 'atresia, intrahepatic biliary':ab,ti OR 'biliary atresia, intrahepatic':ab,ti OR 'biliary atresia, extrahepatic':ab,ti OR 'atresia, extrahepatic biliary':ab,ti OR 'extrahepatic biliary atresia':ab,ti OR 'idiopathic extrahepatic biliary atresia':ab,ti OR 'familial extrahepatic biliary atresia':ab,ti	7,270
#3.	day:ab,ti	1,758,013
#2.	'surgery age':ab,ti	1,534

#1. age:ab,ti 4,141,787

Cochrane Library: a total of 33 articles

ID Search Hits

#1 MeSH descriptor: [Biliary Atresia] explode all trees 45

#2 (Biliary atresia or Atresia, Biliary or Intrahepatic Biliary Atresia or Atresia, Intrahepatic Biliary or Biliary Atresia, Intrahepatic or Biliary Atresia, Extrahepatic or Atresia, Extrahepatic Biliary or Extrahepatic Biliary Atresia or Idiopathic Extrahepatic Biliary Atresia or Familial Extrahepatic Biliary Atresia):ti,ab,kw (Word variations have been searched) 129

#3 #1 or #2 129

#4 MeSH descriptor: [Portoenterostomy, Hepatic] explode all trees 16

#5 (Portoenterostomy, Hepatic or Hepatic Portoenterostomy or Hepatic Portoenterostomies or Portoenterostomies, Hepatic or Hepatoportoenterostomy or Hepatoportoenterostomies or Kasai Procedure):ti,ab,kw (Word variations have been searched) 48

#6 #4 or #5 48

#7 #3 and #6 46

#8 ("day"):ti,ab,kw (Word variations have been searched) 421836

#9 ("age"):ti,ab,kw (Word variations have been searched) 771637

#10 #8 or #9 961536

#11 #6 and #10 33

CNKI: a total of 1339 articles

((主题%='胆道闭锁') OR (篇关摘=胆道闭锁+先天性胆道闭锁+先天性胆管闭锁+先天胆道闭锁+胆道闭锁症+胆管闭锁)) AND ((主题%='肝门空肠吻合术') OR (篇关摘=肝门空肠吻合术+葛西手术)) AND ((篇关摘=年龄) OR (篇关摘=日龄))
