# Supplementary material for: Impact of early Kasai portoenterostomy on short-term outcomes of biliary atresia: A systematic review and meta-analysis
Source: Front Surg. 2022 Sep 1;9:924506. doi: 10.3389/fsurg.2022.924506 (PMC9475174; doi:10.3389/fsurg.2022.924506)

The sensitivity analysis for JCR of patients with age 60 days or younger and patients with age 61 to 90 days

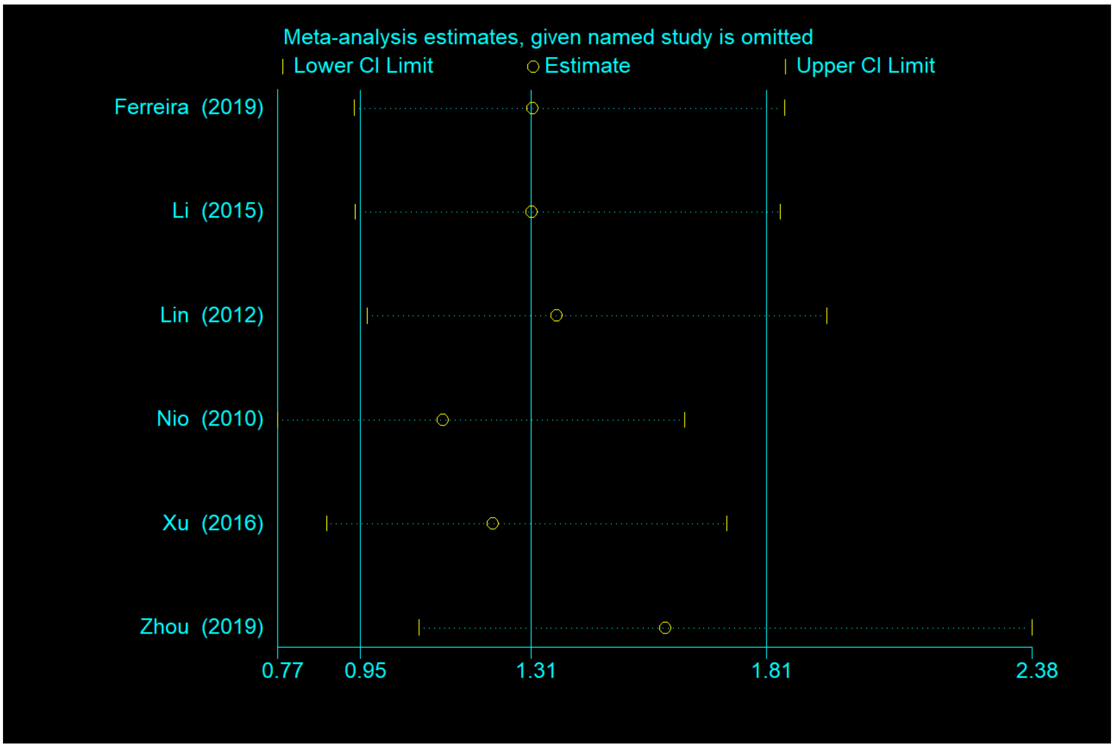

The sensitivity analysis for JCR of patients younger than 90 days and patients older than 91 days

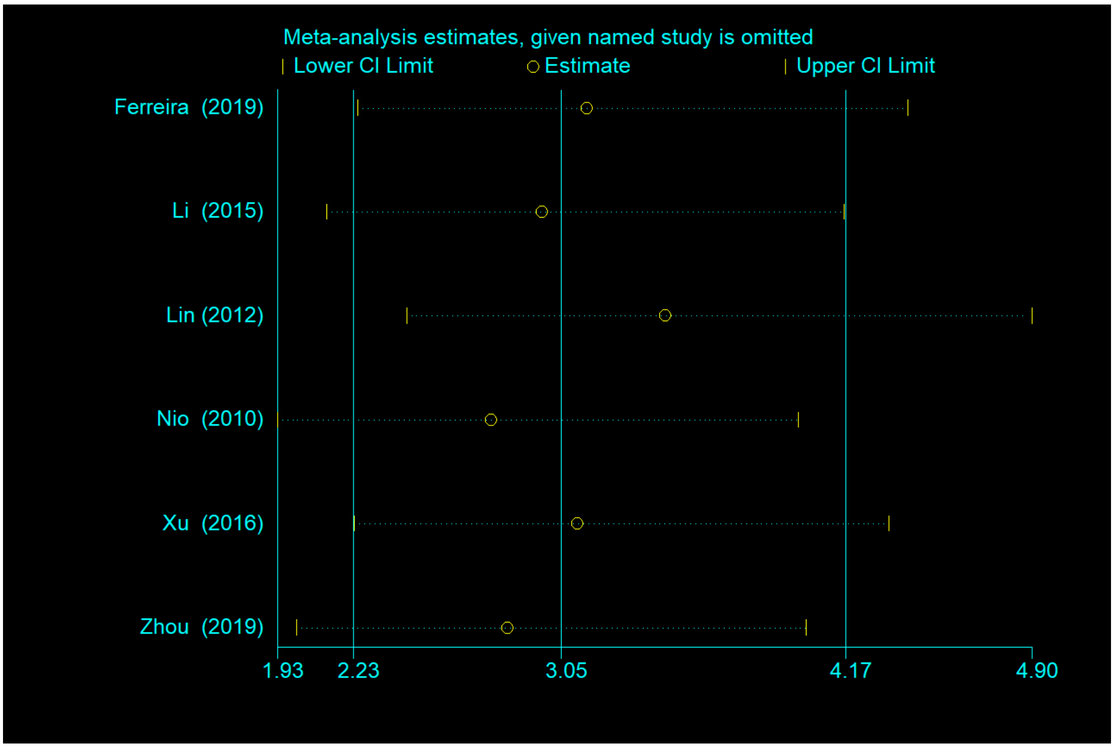

The sensitivity analysis for NLSR of patients younger than 60 days and patients with 61 to 90 days of age

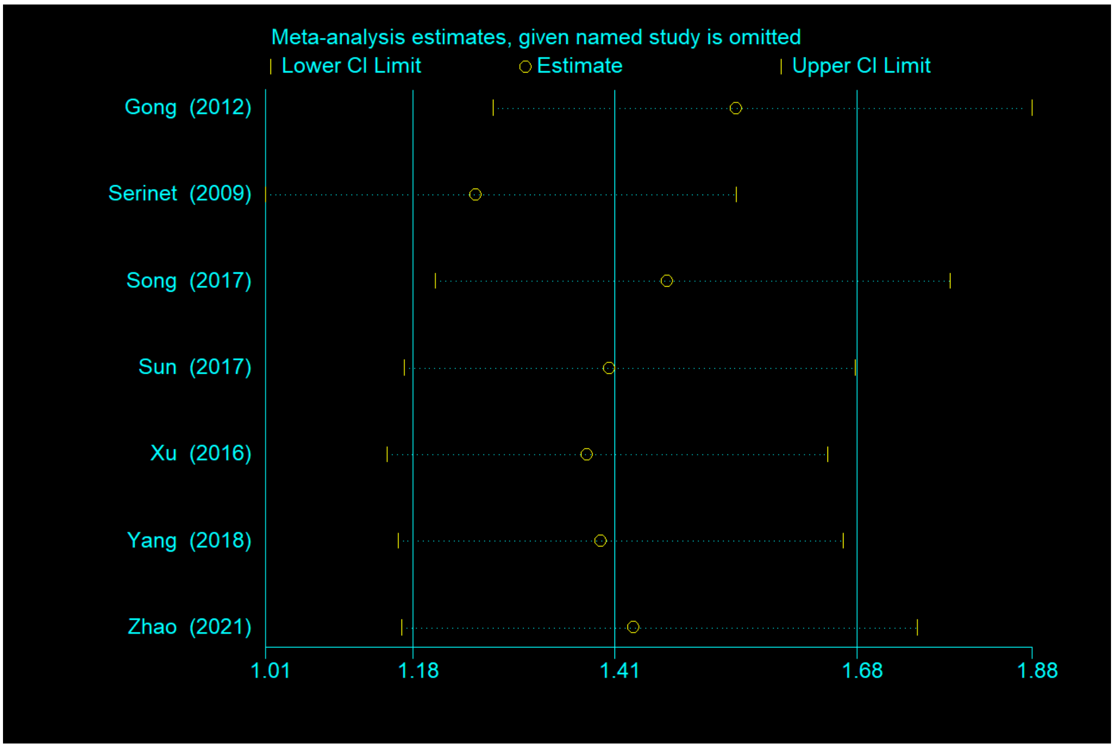

Supplement: Supplementary file 2 [file Data_Sheet_2_v1.pdf]
