# Supplementary material for: Impact of early Kasai portoenterostomy on short-term outcomes of biliary atresia: A systematic review and meta-analysis
Source: Front Surg. 2022 Sep 1;9:924506. doi: 10.3389/fsurg.2022.924506 (PMC9475174; doi:10.3389/fsurg.2022.924506)
Supplement: Supplementary file 3 [file Data_Sheet_3_v1.pdf]

Quality assessment of included the cohort studies according to Newcastle-Ottawa Quality Assessment Scale (NOS)

Quality assessment of cohort studies

**Reference**	**Selection**				**Comparability**	**Outcome**			**Scores**
	Representativeness of the Exposed Cohort	Selection of the Non-Exposed Cohort	Ascertainment of Exposure	Outcome of Interest Was Not Present at Start of Study	Comparability of Cohorts	Assessment of Outcome	Was Follow-Up Long Enough for Outcomes to Occur	Adequacy of Follow Up of Cohorts	
Serinet et al (18).	*	*	*	*	*	*	*	*	8
Nio et al (20).	*	*	*	*	*	*	*	NA	7
Ferreira et al (19).	*	*	*	*	*	NA	*	NA	6
Gong et al (12)	*	*	*	NA	*	NA	*	*	6
Lin et al (21)	*	*	*	*	*	NA	NA	*	6
Li et al (22)	*	NA	*	*	*	*	*	NA	6

Xu et al (23).	*	*	*	*	*	NA	NA	*	6
Song et al (24).	*	*	*	NA	*	*	NA	*	6
Sun et al (25).	*	NA	*	NA	*	*	*	*	6
Yang et al (26).	*	NA	*	*	*	*	*	NA	6
Zhou et al (13).	*	*	*	NA	**	NA	*	*	7
Ryuji et al (27).	*	*	*	NA	**	*	*	*	8
Wu et al (28).	*	*	*	*	*	NA	*	NA	6
Zhao et al (29).	*	*	*	NA	*	*	*	*	7
